# Simultaneous detections of *Olenecamptusbilobus* (Fabricius, 1801) (Cerambycidae, Dorcaschematini) in Europe

**DOI:** 10.3897/BDJ.11.e114432

**Published:** 2023-12-01

**Authors:** Enrico Ruzzier, Carlos R. de Queros, Hugo Mas, Andrea Di Giulio

**Affiliations:** 1 Department of Science, Roma Tre University, Rome, Italy Department of Science, Roma Tre University Rome Italy; 2 NBFC, National Biodiversity Future Center, Palermo, Italy NBFC, National Biodiversity Future Center Palermo Italy; 3 World Biodiversity Association Onlus, Verona, Italy World Biodiversity Association Onlus Verona Italy; 4 Associazione Romana di Entomologia, Roma, Italy Associazione Romana di Entomologia Roma Italy; 5 Laboratori de Sanitat Forestal, Vaersa-Generalitat Valenciana, Quart de Poblet (Valencia), Spain Laboratori de Sanitat Forestal, Vaersa-Generalitat Valenciana Quart de Poblet (Valencia) Spain

**Keywords:** biodiversity, Greece, introduction, longhorn beetles, non-native species, Spain, wood borer

## Abstract

**Background:**

Europe has a long history of non-native species introductions given its central role in global trade in recent centuries. Currently, approximately two hundred cerambycid species have been found in Europe, as the result of introductions between and within biogeographical regions; still, despite better monitoring and stronger restrictions, the arrivals and spread of non-native Cerambycidae continue.

**New information:**

The aim of this contribution is to report and discuss the first European records of the non-native longhorn beetle *Olenecamptusbilobus* (Fabricius, 1801) on the basis of three specimens recorded almost simultaneously in Spain and Greece, respectively.

## Introduction

The introduction of non-native wood-boring beetles is a major phytosanitary concern worldwide (Evans and Oszako 2007, Avtzis and Lakatos 2021), with Cerambycidae (Coleoptera, Chrysomeloidea) considered amongst the most threatening groups (Eyre and Haack 2017, Haack 2017). Introductions and establishment of these beetles are constantly increasing despite strict regulations (Allen et al. 2017), specific monitoring activities and the implementation of new early detection tools and technologies (Rassati et al. 2015, Rassati et al. 2018, Poland and Rassati 2018, Cavaletto et al. 2020, Ruzzier et al. 2021); in addition, several species would appear to be able to evade phytosanitary controls at major entry ports, thus coming to be found already in the wild, often with adventive populations (e.g. Drumont et al. (2014), Maestre del Peral and Bahillo de la Puebla (2017)). In this context, the support provided by non-professional entomologists and citizen science can, in some cases, help to bridge the chronic gap of consistently and extensively monitoring non-native species at the national and/or continental level (see Seidel et al. (2021)). Given this condition, the free and rapid sharing of information on non-native species, such as status, distribution and biological notes in invaded areas, are key building blocks to prevent further invasions (Kenis et al. 2007). Europe, given its importance and central role in the world economy for at least the past three centuries, has a long history of non-native cerambycidae introductions (e.g. Cocquempot and Lindelöw (2010), Lupi et al. (2013), Rassati et al. (2016), Maestre del Peral and Bahillo de la Puebla (2017), Binazzi et al. (2019), Arias and Torralba-Burrial (2020), Ruzzier et al. (2020), Ruzzier et al. (2023)). In recent decades, this trend of introduction has been shifting mainly towards species of East Palearctic and Oriental origin with some capable of developing adventitious populations on the European territory (e.g. Drumont et al. (2014), Sarto i Monteys and Torras i Tutusaus (2018), Russo et al. (2020)) or even undertaking phases of range expansion (Keszthelyi et al. 2019, Lupi et al. 2023). During the summer of 2023, the eastern Asian species *Olenecamptusbilobus* (Fabricius, 1801) (Cerambycidae, Lamiinae, Dorcaschematini) was fortuitously and independently recorded in Spain and Greece, respectively, representing the first case of introduction of this species on the European territory. The nature and importance, as well as the phytosanitary relevance of these findings, are discussed in this paper.

## Materials and methods

*Olenecamptusbilobus* specimens from Les Salades (Elche, Spain) were collected at night in the same spot, in two different occasions, while standing on a white wall and most probably attracted by the light of a street lamp. Both specimens are stored in Carlos R. de Queros private collection (Scandicci, Florence - Italy). The record from Lemnos Island was retrieved from INaturalist. All findings were notified to the local phytosanitary services.

## Taxon treatments

### Olenecamptus bilobus

(Fabricius, 1801)

708FDC5C-CF97-5A5A-87C7-713C1027F409

https://www.gbif.org/species/9191189

https://doi.org/10.1080/23802359.2021.1875897

#### Materials

- **Type status:** Other material. **Occurrence:** individualCount: 1; lifeStage: adult; establishmentMeans: non-native; occurrenceStatus: adventive; occurrenceID: 5647E75F-3C2C-5B66-80CF-A3831563BF20; **Taxon:** scientificName: Olenecamptusbilobus (Fabricius, 1801); order: Coleoptera; family: Cerambycidae; genus: Olenecamptus; specificEpithet: *bilobus*; taxonRank: species; scientificNameAuthorship: (Fabricius, 1801); nomenclaturalCode: ICZN; **Location:** continent: Europe; country: Spain; countryCode: SP; stateProvince: Alicante; county: Elche; locality: Les Salades; decimalLatitude: 38.305881; decimalLongitude: -0.643427; geodeticDatum: WGS84; **Identification:** identifiedBy: Enrico Ruzzier; **Event:** eventDate: 2023-06-01
- **Type status:** Other material. **Occurrence:** individualCount: 1; lifeStage: adult; establishmentMeans: non-native; occurrenceStatus: adventive; occurrenceID: 6D500A5D-145D-5BA1-9308-382731F02565; **Taxon:** scientificName: *Olenecamptusbilobus* (Fabricius, 1801); order: Coleoptera; family: Cerambycidae; genus: Olenecamptus; specificEpithet: *bilobus*; taxonRank: species; scientificNameAuthorship: (Fabricius, 1801); nomenclaturalCode: ICZN; **Location:** continent: Europe; island: Lemnos; country: Greece; countryCode: GR; stateProvince: Lemnos regional unit; county: Moudros; decimalLatitude: 39.878321; decimalLongitude: 25.273639; geodeticDatum: WGS84; coordinateUncertaintyInMeters: 52; **Identification:** identifiedBy: Enrico Ruzzier; **Event:** eventDate: 2023-08-02; **Record Level:** basisOfRecord: HumanObservation; source: https://www.inaturalist.org/observations/176326999
- **Type status:** Other material. **Occurrence:** individualCount: 1; lifeStage: adult; establishmentMeans: non-native; occurrenceStatus: adventive; occurrenceID: 918B9A3A-AA4A-51EA-B006-B088FD403FA7; **Taxon:** scientificName: *Olenecamptusbilobus* (Fabricius, 1801); order: Coleoptera; family: Cerambycidae; genus: Olenecamptus; specificEpithet: *bilobus*; taxonRank: species; scientificNameAuthorship: (Fabricius, 1801); nomenclaturalCode: ICZN; **Location:** continent: Europe; country: Spain; countryCode: SP; stateProvince: Alicante; county: Elche; locality: Les Salades; decimalLatitude: 38.305881; decimalLongitude: -0.643427; geodeticDatum: WGS84; **Identification:** identifiedBy: Enrico Ruzzier; **Event:** eventDate: 2023-09-16

#### Distribution

**Figure 1.**
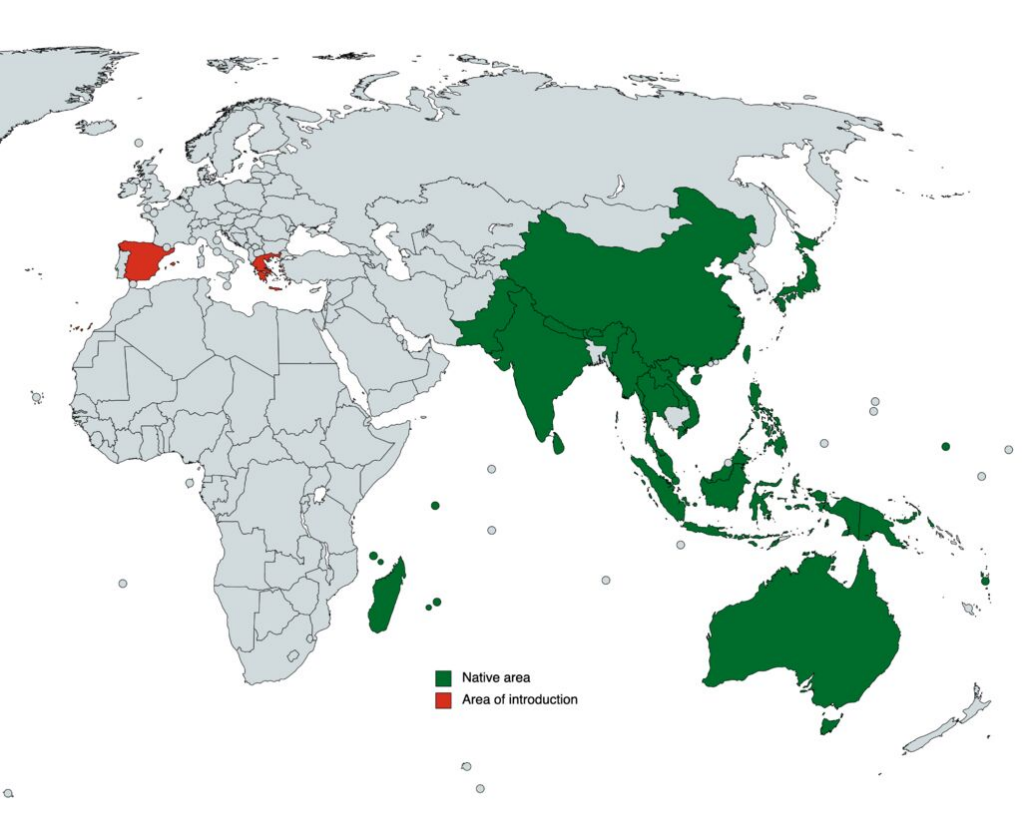
Distribution map of *Olenecamptusbilobus* (Fabricius, 1801).

*Olenecamptusbilobus* is widely distributed throughout the Australasian, Eastern Palearctic, Oriental Region and Madagascar (TITAN database) (Fig. 1). The data here provided represent the first records of this species in Europe.

## Discussion

The discovery of this species further highlights the need for attention to the accidental introduction of non-native species into the European territory and further highlights the issue regarding the capability of some beetles to systematically evade controls.

### Nature of the findings and introduction pathway

**Figure 2.**
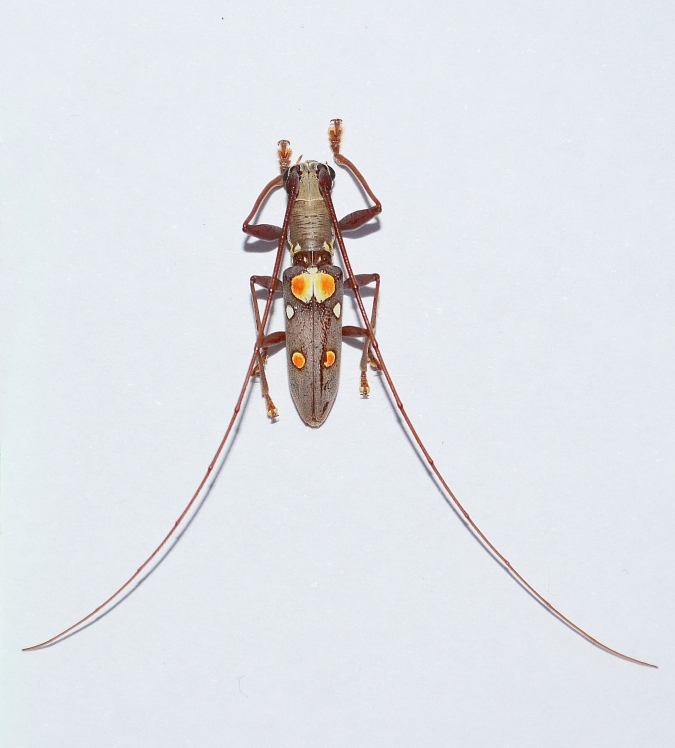
*Olenecamptusbilobus* (Fabricius, 1801) recorded in Les Salades - Elche, Spain (photo credit: CR. de Queros).

**Figure 3.**
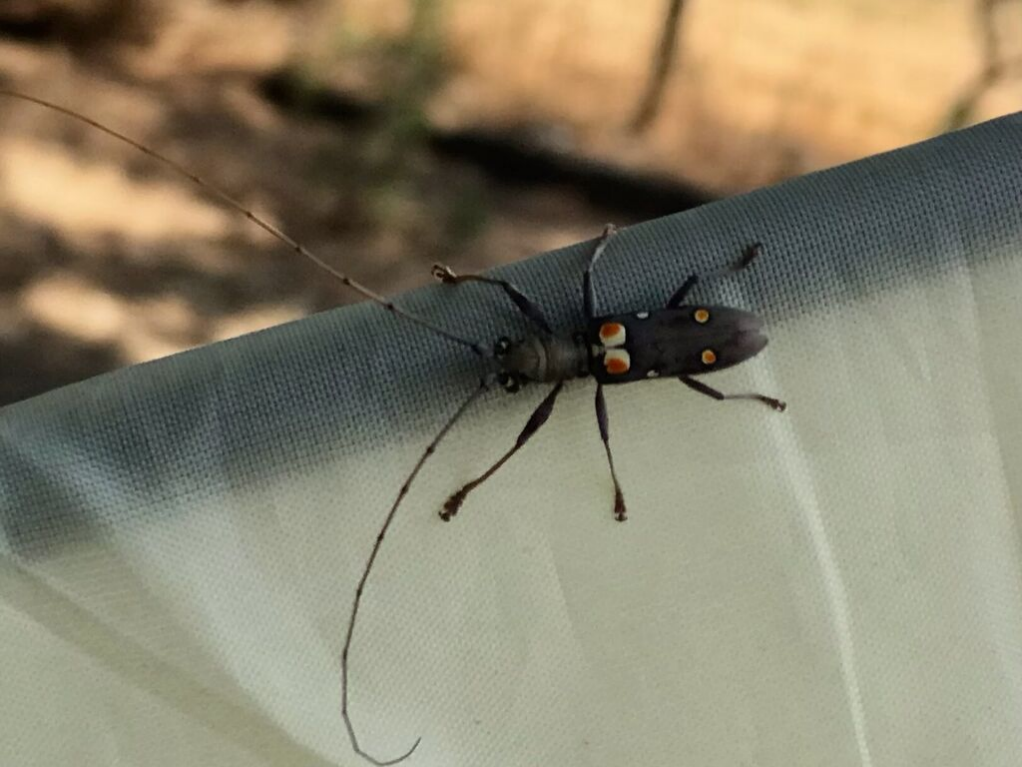
*Olenecamptusbilobus* (Fabricius, 1801) from Lemnos Island, Greece (Source: INaturalist; photo credit: Alexandros Galanidis).

*Olenecamptusbilobus* consists of multiple subspecies, separated primarily on the basis of the chromatic patterns of elytra (see Dillon and Dillon (1947)). The three specimens seem, in their general features, to belong to the same taxon (Figs 2, 3). However, the extreme chromatic variability of the species complicates the attribution of these specimens to one of the known subspecies, thus making it impossible to state with certainty their precise origin. The accidental introduction of *O.bilobus* on the island of Lemnos (Greece) is mostly attributable to naval traffic; it is, in fact, widely recognised that naval transport is the main pattern for the induction of non-native wood-boring beetles (Meurisse et al. 2019); however, given the short distance between the discovery area and the local airport (~ 5.5 km), it is not possible *a priori* to exclude transport by air. Similarly, the specimens collected in Les Salades (Elche, Spanish mainland), were collected not at great distances from Alicante's airport and port (~ 7.5 and ~ 13 km, respectively), an area where other non-native beetles were found in previous monitioring activities (Gallego et al. 2022, Mas et al. 2023). In both cases, however, the fact that the specimens were recorded in areas hosting plant nurseries would seem to suggest the import of non-native plants for ornamental purposes as the most plausible vector of introduction; this condition has already been noted for other non-native Cerambycidae to Europe (Cocquempot 2006, Cocquempot and Lindelöw 2010). In fact, since *O.bilobus* develops primarily on live plants or parts of a plant that have recently died and are still humid (Hanks 1999), it is very unlikely that it might have been introduced with processed wood. It remains, however, unclear if these beetles represent sporadic introductions or locally adventive populations in their early phases.

### Phytosanitary relevance

*Olenecamptusbilobus* is recognised as a species of modest to normal phytosanitary interest in its countries of origin, both at the larval and adult stages (Stebbing 1914, Duffy 1968, Waterhouse 1993, Kariyanna et al. 2017, Balikai et al. 2022, Kallekkattil and Mani 2022, Mani 2022); the species is included, without any further information, in the EPPO Database and in the CABI Compendium, while it is not in any European alert or quarantine list. The life cycle of the species was investigated under laboratory conditions by Khan and Maiti (1982), while in nature, larvae generally bore into the sapwood in their early instars and subsequently penetrate into the hardwood (Mathew 1982, Khan and Maiti 1983); adults are generally less impacting gnawing the green bark of shoots or chewing large leaves (Duffy 1968). It is not clear whether the species prefers live or dead plants for oviposition; records seem to suggest that it is capable of developing in both (Beeson 1941), although a certain degree of humidity of dead wood is essential to ensure its survival (Khan and Maiti 1983). The species is polyphagous, but it seems to prefer plants of the genera *Arthrocarpus*, *Ficus* and *Morus* (Moraceae) (Beeson 1941, Duffy 1968, Kariyanna et al. 2017). Currently, there is no specific measure for its control other than the elimination of adults and the destruction of affected plants. The species can be collected at light (Sreedevi et al. 2016) and it has been recorded responding to a multi-lure blend composed of pheromones and host volatiles in its native environment (Roques et al. 2023). To date, a pest categorisation is needed to estimate the impact of this species once established on the areas of arrival.

## Supplementary Material

XML Treatment for Olenecamptus bilobus
